# Salivary Testosterone and Cortisol Levels in Tunisian Elderly Male Patients With Mild Alzheimer’s Disease. Implications of Musical Therapy And/Or Physical Rehabilitation

**DOI:** 10.3389/fphys.2022.839099

**Published:** 2022-08-05

**Authors:** Sarah Chéour, Chouaieb Chéour, Chiraz Kilani, Aymen Guemri, Dawser Zineddine, Riadh Khélifa, Rashmi Supriya, Nicola Luigi Bragazzi, Foued Chéour, Julien S. Baker, Sabri Gaied-Chortane

**Affiliations:** ^1^ High Institute of Sport and Physical Education of Ksar Said, Manouba, Tunisia; ^2^ High Institute of Sport and Physical Education of Sfax, Sfax, Tunisia; ^3^ High Institute of Education and Continuous Training of Tunis, Tunis, Tunisia; ^4^ Faculty of Economics and Management of Sfax, Sfax, Tunisia; ^5^ Centre for Health and Exercise Science Research, Hong Kong Baptist University, Kowloon Tong, Hong Kong SAR, China; ^6^ Laboratory for Industrial and Applied Mathematics (LIAM), Department of Mathematics and Statistics, York University, Toronto, ON, Canada

**Keywords:** alzheimer disease, elderly male patients, music therapy, physical rehabilitation, testosterone, cortisol

## Abstract

Changes in salivary testosterone (T) and cortisol (C) levels were assessed in elderly Tunisian male patients with mild Alzheimer’s disease (AD) subjected to music therapy and/or physical rehabilitation. Male patients with mild AD (N = 26; age = 76.23 ± 4.27 years; weight: 74.76 ± 5.36 kg) were randomly assigned into four groups for three 60-min sessions per week for 4 months; including Group1 or control group (Co) (n = 6); Group2 (n = 6), participated in physical rehabilitation (PR); Group3 (n = 7), subjected to music therapy (MT) and Group4 (n = 7), participated simultaneously in music therapy and physical rehabilitation (MT + PR). Salivary T levels increased (ηp^2^ = 0.7) and C levels decreased (ηp^2^ = 0.69), significantly (*p* < 0.001) in the PR, MT and MT + PR groups compared to the Co group respectively. Also, increases in salivary T levels and decreases in C levels in MT + PR group were greater compared to the other groups. MT increased T levels (*p* < 0.001) and decreased C levels (*p* < 0.05) to a greater extent than the PR group respectively. Changes in salivary T levels were positively (r = 0.83; *p* < 0.001) and C levels were negatively (r = -0.86; *p* < 0.001) correlated in the PR, MT and MT + PR groups with changes in MMSE in AD patients. This study highlights that combination of MT and PR holds potential to treat AD.

## Introduction

Alzheimer’s disease (AD) is a cognitive disease. AD is the principal pathology in the world displaying negative impacts on both the health and social ability of patients while inducing considerable economic costs (Dauphinot et al., 2022). AD is one of the greatest health challenges of this century for humanity (DeTure and Dickson, 2019; Klatt et al., 2019; Eftychios et al., 2021). AD is a progressive and devastating neurodegenerative disorder of the brain most often characterized by loss of neurons and synapses. These losses are particularly in regions related to memory and cognition which can ultimately affect behaviour, speech, visuospatial orientation, and the motor system. AD is the most common form of dementia, and its prevalence increases dramatically with age (Picanco et al., 2018; Takeda, 2019a).

A growing body of evidence suggests that an abnormal level of certain steroid hormones including testosterone (T) and cortisol (C) are correlated with the main features of AD pathogenesis, in particular β-amyloid deposition, tau hyperphosphorylation, alongside synaptic deficits and cognitive disorders, disturbance of memory, mind and mood, depression, etc. in animal models and human patients (Laske et al., 2009; Wang et al., 2018; Ouanes and Popp, 2019). T and C are also associated with structural aspects of the hippocampus and its related cognitive abilities (Panizzon et al., 2018; DeTure and Dickson, 2019). Indeed, the interactive effects between T and C for hippocampal volume and episodic memory performance in men have been reported previously (Fukui et al., 2012; Panizzon et al., 2018). In both sexes, sex hormone levels decrease with aging (Fukui et al., 2012). Higher T levels are associated with larger hippocampal volume and better memory performance even in a neurotoxic hormonal environment associated with high C levels (Fukui et al., 2012; Panizzon et al., 2018; Eftychios et al., 2021). The differences in T production between men and women might explain the higher prevalence of the disease in women (Picanco et al., 2018). T is a steroid hormone secreted by the Leydig cells of the testes under hypothalamic and pituitary control defining the hypothalamo-pituitary-testicular (HPT) axis and in small amounts from the adrenal cortex (Bouazizi et al., 2019). In both sexes, T follows a diurnal rhythm with peak concentrations occurring in the morning followed by a progressive decline over the course of the day, rising again at night during sleep (Bouazizi et al., 2019). Many studies have also reported a link between low T levels and the risk of developing AD which is the leading cause of dementia (Lei and Renyuan, 2018). Thus, T is increasingly considered as an effective biomarker confirming the prevalence of AD in humans (Kaloni and Negi, 2019). C is a corticosteroid hormone released according to circadian rhythms with peak concentrations in the morning. The hormone is produced by the adrenocortical glands under hypothalamic and pituitary control defining the hypothalamic-pituitary-adrenal (HPA) axis. Abnormal levels of C have been negatively associated with structural aspects of the hippocampus, related to the main features of AD pathogenesis such as β-amyloid deposition, tau hyperphosphorylation, and manifestations of synaptic deficits and cognitive impairment (Toledo et al., 2011; Notarianni, 2013; Wang et al., 2018; Ouanes and Popp, 2019). Higher cortisol levels have also been reported to be associated with depressive symptoms (Balardin et al., 2011).

Music therapy (MT) is the most common non-pharmaceutical treatment for AD (Eftychios et al., 2021). The effectiveness of MT can depend on the quality and length of treatment as well as other factors (Leubner and Hinterberger, 2017). Because of the wide and heterogeneous range of applications, there might be some direct influence on the results of MT for AD including listening to music, chants, music-based interventions, background music, music with activities and multisensory stimulation (Leggieri et al., 2019). MT improves social behaviour by reducing wandering, restlessness, and agitated behaviours. The most common effects of MT are improved social behaviors, such as interpersonal interactions and conversations (Cuddy et al., 2012; Fang et al., 2017). Music reaches almost every area of the brain, which is not surprising since music makes individuals rhythmically aware, encourages recall of vivid memories, and has the potential to lighten the mood (Fang et al., 2017; Eftychios et al., 2021).

Traditionally, studies on the efficacy of MT in patients with AD have focused on changes in symptoms such as dementia most typically and other problematic behaviours such as aggressive behaviors, depression, disturbance of mood, and decreased sociality (Groene, 1993; Lord and Garner, 1993; Hanser and Thompson, 1994). Fukui et al. (2012) conducted a study investigating the verification of the effects of MT on the variation of T in Alzheimer’s patients. The study reported that MT increases T levels in AD patients. The findings of the study have never been invalidated or confirmed. However, there are no similar studies that have investigated C concentrations in this population.

The effectiveness of physical exercise in preventing disability risks and cognitive and memory decline have been well reported (Picanco et al., 2018; Kaloni and Negi, 2019; Marques et al., 2019). Physical activity can also improve brain function and memory and may delay the decline in the ability to perform tasks in people who have AD by improving their strength, balance, walking ability, etc. Moderate exercise has been shown to help increase T and decrease C levels in older populations (Blackwood and Martin, 2017; Dawson et al., 2019; Klatt et al., 2019).

As mentioned above both MT and PR can improve brain function and may help people to recover from AD. However, study designs to observe the combined effect of MT and PR on mild AD patients using salivary testosterone and cortisol levels for evaluation of cognitive function and memory capacity has been lacking. Advanced-stage AD patients suffer from a problem of major motor dysfunction and this disadvantages the application of a physical rehabilitation program for these populations. Also, AD patients suffer with episodic disorders and have a problem in daily functioning. Therefore, in this study we recruited mild AD subjects diagnosed as being at the moderate stage. This was done because at this stage there are chances of partially reversing the disease.

The objectives of this study were to evaluate MT and PR effects used as a single or combined intervention on cognitive function and memory capacity evaluated by the MMSE. A further aim was to examine salivary T and C levels in elderly Tunisian male patients with mild AD following 4 months of intervention three times a week for a duration of 60-min.

## Materals and Methods

### Participants

This clinical study was undertaken at the Neurology Department of the University Hospital Center (UHC) of Monastir, Tunisia, during the months of February, March, April, and May 2021. The patients were recruited from all areas surrounding Monastir and Sousse, Tunisia. All patients were screened based on eligibility criteria prior to commencement of the study. Only male subjects who had provided written informed consent and met the eligibility criteria were included in this study. The study was completed using a sample of 26 elderly Tunisian male patients who were carefully selected.

### Eligibility Criteria

We only selected male patients aged 60 and over (age: 76.23 ± 4.27 years; weight: 74.76 ± 5.36 kg) who had Mini-Mental State Examination (MMSE) scores of at least 19 (MMSE: 19.82 ± 0.4). All participants in this study had a homogeneous education level (years: 7.6 ± 2.1) and were at the early stage of the disease and presented with mild cognitive impairment. During the study, patients were always accompanied by a spouse, child, close friend, etc. to encourage them and/or to facilitate their understanding of the study protocol. Only patients available to be involved in the study were selected to conduct the research. Patients with head trauma, late-stage disease, consuming drugs that may affect metabolism and the production of steroid hormones, who have no acceptable hearing senses, showing injury or any type of fracture, were excluded from the study. Furthermore, patients with obesity, metabolic diseases or smokers were also excluded from the study. Final decision of inclusion was determined jointly by a neurologist, neuropsychologist, and the researchers leading the project.

### Legal and Ethical Aspects

Prior to commencement of the study, permission was obtained and granted by the Committee for the Protection of Persons (CPP) of the Monastir region, Tunisia. The purpose of the committee is to judge the scientific quality of projects and to ensure the safety of participants (CEM131/19). The study presented here was designed well and did not impinge on the integrity of the patients or pose any particular risk to participants and was therefore approved by the committee. Patients were always informed before each rehabilitation session of the research protocol and the objectives of the study. Our study followed CONSORT statement.

### Procedures

Male patients underwent MT and PR as a single or combined intervention or served as a control. They were randomly assigned into four groups. The first, which included six patients, served as a control group (Co). The second, including six patients, participated in physical rehabilitation (PR). The third, consisting of seven patients, and was subjected to music therapy (MT). The last group, comprising of seven patients, was subjected simultaneously to music therapy and physical rehabilitation (MT + PR). Participants were required to participate in the study for 4 months (16 weeks) for three sessions per week with a duration of 60-min per session.

The evaluation of the levels of cognitive and mental function, memory capacity and intellectual aptitude of the patients was carried out using the Mini-Mental State Examination (MMSE) test (Folstein et al., 1975).

Remediation of AD patients was performed using MT and/or PR. Musical stimulation was based on listening to music chosen for patients belonging to the MT and MT + PR groups with musical extracts selected by a music therapist from a repertoire of traditional Tunisian music. The songs were chosen from the Tunisian musical heritage of the 60, 70 and 80 s. The singers were Ali RIAHI, Hédi JOUINI, Sadok THRAYA, Oulaya, Naama, Saliha. In relation to the combined use of music therapy and physical rehabilitation (MT + PR): Patients underwent physical rehabilitation while simultaneously listening to music for 1 hour. They did the exercises while listening to the music. A systematic review and meta-analysis found that music therapy involving listening to music was more effective in reducing behavioural symptoms compared to active music therapies (Tsoi et al., 2018). Listening to music after 6 months has been reported to improve cognitive abilities in patients with mild AD. Music listening is used to stimulate verbalization, memories, or to encourage relaxation (Eftychios et al., 2021). It should be noted that prior to starting the study, we inquired about individual musical interests and preferences, and any artists and songs that may have provided some positive moments in the patients’ personal lives. The emotions that music can provide are important, especially when linked to autobiographical memories. The music used in this study was facilitated via our personal computer equipped with speakers. It should be noted that the MT + PR group was always subjected simultaneously to MT and PR at each remediation session.

The patients belonging to the PR and MT + PR groups were subjected to muscle strengthening exercises and joint movements adapted for the upper and lower limbs according to an established program (Maltais et al., 1996). Physical rehabilitation included 20 min of walking, strengthening muscles and joint movements, 20 min of balancing and posture, and 10 min of stretching and calming the body. It should be noted that each rehabilitation session was always preceded by a warm-up training session for 10 min which corresponded to walking on a treadmill at a very low speed (5 km/h) to avoid the risk of injury.

### Saliva Collection and Analysis

Hormones were assayed in saliva. Saliva collection is non-invasive and requires minimum equipment and expertise to obtain meaningful data. Using a passive drool method, saliva samples (at least 1 ml) for the measurements of salivary T and C levels were obtained from all subjects at 9 a.m. via expectoration into graduated 2 ml cryovials (Salimetrics, State College, PA). Data collection times were standardized to control potential diurnal variation effects. Samples were centrifuged and placed immediately into a refrigerator and frozen at −20°C for subsequent analysis. Samples were assayed in duplicate (without separation or extraction) for T (pmol/L) and C (nmol/L) using protocols from commercially available immunoassay (Salimetrics, State College, PA). All samples were assayed for T using a high-sensitivity enzyme immunoassay (EIA) with a lower limit of sensitivity of 2.3 pmol/L, and average intra- and inter-assay coefficients of variation less than 7.3 and 9.4%, respectively. Saliva samples were assayed for C using EIA with a lower limit of sensitivity of <0.026 nmol/L, and average intra and inter-assay coefficients of variation 4.34 and 7.90%, respectively. Patients using prednisolone or other corticosteroids were excluded. Patients were instructed not to eat, drink, or smoke in the 30 min prior to saliva collection. The exact collection times were recorded by a member of the research team. We did not lose any samples in the study.

### Statistical Analysis

Data are presented as mean ± standard deviation (SD). Statistical analysis of the data as well as verification of normality was performed using the Shapiro-Wilks test due to the small number of patients. Based on a normal distribution, parametric and non-parametric tests were used. Comparisons between groups were investigated using Student’s *t* test or Mann-Whitney *U* test, according to the characteristics of the data. One-way repeated measures analysis of variance (ANOVA) and Bonferroni’s post hoc test was used to compare the effects of MT and PR as a single or combined intervention prior and post intervention. To allow a better interpretation of the results, the effect sizes were calculated as partial eta-squared (ηp^2^) for the ANOVA analysis to determine the magnitude of the change. Pearson correlation coefficient was also used to measure the linear association between the changes in salivary T and C levels with changes in MMSE in AD patients (Snedecor and Cochran, 1957). All statistical analysis was carried out using the commercial statistical package software for social sciences (SPSS version 23.0, IBM, United States) and MedCalc Statistical software version 17.9.7 (MedCalc bvba software, Ostend, Belgium; http://www.medcalc.org; 2017). A significance level of *p* ≤ 0.05 was used for all analyses.

## Results

PR, MT and MT + PR significantly improved cognitive function and memory capacity (MMSE) of Tunisian elderly male patients with mild AD compared to the Co (*p* < 0.001) after a 4-months intervention for three 60-min sessions per week. However, this improvement was greater in the MT + PR (MMSE score: 24.58 ± 1.97) group than in MT (MMSE score: 22.97 ± 1.81) and PR (MMSE score: 21.33 ± 1.67) groups. MT improved cognitive function and memory capacity of patients to a greater extent than the PR (*p* < 0.05). The MMSE level decreased significantly in the Co group at the end of the study (MME score: 16.16 ± 2.41) (*p* < 0.001).

**FIGURE 1:**
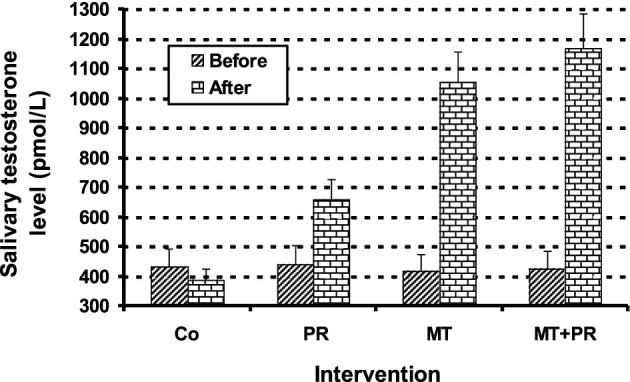
Evolution of salivary T levels of Tunisian elderly male patients with mild AD in Co, PR, MT and MT + PR groups after 4 months of intervention at the rate of three sessions of 60-min per week. The results correspond to mean ± standard deviation for 6-7 patients. Intervention: Co (control), PR (physic rehabilitation), MT (music therapy) and MT + PR (music therapy + physic rehabilitation).

Figure 1 shows the salivary T levels of Tunisian elderly male patients with mild AD for Co, PR, MT and MT + PR groups after 4 months of intervention for three 60-min sessions per week. Salivary T levels increase significantly in the PR, MT and MT + PR groups at the end of the intervention period, unlike the Co group Co (ηp^2^ = 0.7; *p* < 0.001). However, this increase was greater for MT + PR group. MT increased salivary T levels more than PR (*p* < 0.001). Changes in salivary T levels in PR, MT and MT + PR groups were positively correlated with changes in MMSE in AD patients (r = 0.83; *p* < 0.001).

**FIGURE 2:**
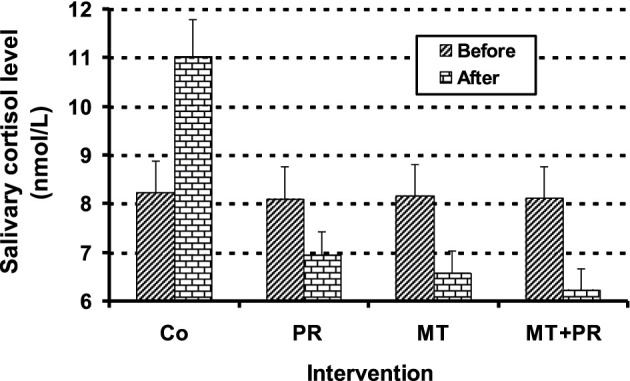
Evolution of salivary C levels of Tunisian elderly male patients with mild AD in Co, PR, MT and MT + PR groups after 4 months of intervention at the rate of three sessions of 60-min per week. The results correspond to mean ± standard deviation for 6-7 patients. Intervention: Co (control), PR (physic rehabilitation), MT (music therapy) and MT + PR (music therapy + physic rehabilitation).

Figure 2 shows the salivary C levels of Tunisian elderly male patients with mild AD for groups Co, PR, MT and MT + PR after 4 months of intervention. Salivary C levels decreased significantly in PR, MT and MT + PR groups at the end of the rehabilitation period, unlike the Co group (ηp^2^ = 0.69; *p* < 0.001). However, this decrease was greater in MT + PR group than in MT or PR group (*p* < 0.05). Salivary C level decreased more significantly in MT than PR group (*p* < 0.05). Changes in salivary C levels in PR, MT and MT + PR groups were negatively correlated with changes in MMSE in AD patients (r = -0.86; *p* < 0.001).

## Discussion

Changes in T and C levels were assessed among four groups in elderly Tunisian male patients (N = 26; age = 76.23 ± 4.27 years; weight: 74.76 ± 5.36 kg) with mild Alzheimer’s disease (AD) subjected to MT and/or PR. We explored increases in salivary T levels and decreases in C levels in MT + PR group were greater compared to the other groups. MT increased T levels (*p* < 0.001) and decreased C levels (*p* < 0.05) to a greater extent than the PR group respectively. Changes in salivary T levels were positively (r = 0.83; *p* < 0.001) and C levels were negatively (r = -0.86; *p* < 0.001) correlated in the PR, MT and MT + PR groups with changes in MMSE in AD patients. This study highlights that combination of MT and PR shows potential to treat AD.

AD is one of the most common types of dementia affecting the elderly (Ishii and Iadecola, 2015). AD is a progressive multifactorial neurodegenerative brain disorder with no known cause and several alterable and non-alterable risk factors are associated with its development (Takeda, 2019b; DeTure and Dickson, 2019). AD is characterized by neurodegenerative changes associated with a gradual loss in cognitive function, memory, and behavioural changes. Age is the greatest non-genetic risk factor (Rygiel, 2016; Takeda, 2019a,b). AD causes functional as well as structural disturbances of the brain’s nerve cells due to an accumulation of toxic proteins that exhibit clinical syndromes. In the early stages of the disease, it also causes synaptic dysfunction of nerve cells thereby affecting communication within neural circuits which are important for memory and other cognitive functions (Behl et al., 2021).

Our study aimed to evaluate the impact of a non-drug therapy in AD patients. Our goal was to test the non-drug effectiveness combining a cognitive stimulation program based on MT with a PR program on the production of the steroid hormones T and C in Tunisian elderly male patients with mild AD following a 4-months intervention. Music interventions have grown in popularity and have been widely adopted as a potential non-pharmacological therapy for patients with AD to treat cognitive and/or behavioural symptoms of the disease (Leggieri et al., 2019). In spite of the prevalence of such therapies, evidence of their effectiveness has been speculative in the available literature (Leubner and Hinterberger, 2017; Leggieri et al., 2019). Physical therapy also plays an important role in reducing complications of AD. It mainly involves the use of aerobic or anaerobic exercise aimed at improving functional capacity, preventing cognitive decline and memory, reducing the risk of falls during the illness, and reducing medication used although current evidence remains insufficient to conclude that physical therapy is effective for AD (Picanco et al., 2018; Kaloni and Negi, 2019; Marques et al., 2019). Physical activity appears to maintain optimal blood circulation in the brain and reduce the loss of connections that naturally occur at this level with aging. However, the current evidence remains insufficient to conclude that physical therapy is an effective alternative to drug therapies in the treatment of AD (Dawson et al., 2019; Kaloni and Negi, 2019; Marques et al., 2019). We compared four groups of patients, single intervention (i.e., MT or PR), associated intervention (i.e., MT + PR) or control (i.e., Co) after 4 months.

Our study demonstrated that MT and PR used as a single intervention or in combination significantly influences salivary T and C levels in Tunisian elderly male patients with mild AD. Indeed, the interventions induced the production of salivary T and reduced the production of salivary C. The effects of the interventions on the concentrations of these two hormones become even more pronounced if the interventions were used in combination. These variations were significantly correlated with the improvements in patients’ MMSE indicating the importance of these hormones in the control of AD. Induction of T production by MT in AD has been well reported although verification of its effects in association with PR has not been reported (Fukui et al., 2012). Also, to our knowledge, no work has verified its effects on salivary C levels. Our results highlight the beneficial effects of MT and PR on AD mainly if they are used in combination. By increasing salivary T and reducing C levels in Alzheimer’s patients’ variations are correlated with improvements in the MMSE scores in this population. Indeed, different studies have reported associations between hippocampal structure and functions on the one hand, and T and C levels on the other hand in humans (Atwi et al., 2016; Panizzon et al., 2018). It has been reported previously that T promotes hippocampal neurogenesis, regulates synaptic plasticity in the hippocampus and maintains hippocampal volume in animals (Harley et al., 2000; Galea et al., 2006; Toda et al., 2019). T also appears to be neuroprotective with respect to AD-related pathology, by regulating the accumulation of β-amyloid in cultured hippocampal neurons, and by preventing the formation of tau-related pathologies (Papasozomenos and Shanavas, 2002). Studies conducted on humans have shown that T levels consistently correlate positively with hippocampal volume in male and female adolescents as well as with cerebral blood flow within the hippocampus in elderly men **(** Moffat and Resnick, 2007; Neufang et al., 2009; Notarianni, 2013) The androgenic hormone T has been found to have positive associations with hippocampal-mediated cognitive processes in healthy adults, though findings in this area remain mixed (Boss et al., 2014). Lower T levels were associated with poorer memory performance (Lei and Renyuan, 2018; Panizzon et al., 2018). It has also been reported that with increases in T levels, cognitive test score increases in elderly males (Yaffe et al., 2002). Contrary to this, elevated C a glucocorticoid produced by the adrenal cortex glands, has been negatively associated with structural aspects of the hippocampus and its related cognitive abilities. Higher C levels have been reported as associated with depressive symptoms (Chen et al., 1997; Balardin et al., 2011). Such observations may be suggesting that chronic glucocorticoid secretion following stress may contribute to cognitive decline in AD in the elderly **(** Sapolsky et al., 1986). Indeed, activation of the hypothalamic-pituitary-adrenal (HPA) axis causes secretion of glucocorticoids, secreted in the form of C in humans, as a response to stress and for maintaining homeostasis (Notarianni, 2013; Herman et al., 2016; Russell and Lightman, 2019). Thereby, AD-related HPA axis hyperactivity is most frequently observed as elevated peak C levels in the morning although some studies reported no significant differences in morning C levels between AD patients and cognitively normal controls (Wang et al., 2018). However, as with T, findings have at times been inconsistent across both neuroimaging and cognitive studies (Frodl and O’Keane, 2013; Panizzon et al., 2018). Hence, suggesting these two hormones are very important in assessment of Alzheimer’s patients.

Thus, we suggest that the incorporation of music therapy (musical extracts chosen by a music therapist) along with physiotherapy on elderly male patients with mild AD might manage AD and help in alleviating dementia.

Although the adopted sample size resulted in a statistical power of 80% or above for most of our significant outcome measures, the constraint of sample size might also be a limitation in the present study. The main limitation of this study relies on the fact that these data are specific to Tunisian music and subjects from Tunisia. Also, another limitation of this study is that the results and conclusion are specific to male populations.

## Conclusion

We conclude that MT and PR as a combined intervention is more effective than a MT or PR intervention individually for improved cognitive function and memory capacity among Tunisian elderly male patients with mild AD as evaluated by the MMSE. Also, 4 months intervention of either MT, PR or MT + PR (3times/week) for 60 min resulted in favourable changes in T and C levels in elderly Tunisian male patients with mild AD.

## Data Availability

The original contributions presented in the study are included in the article/Supplementary material, further inquiries can be directed to the corresponding author.
